# A meta-approach for improving the prediction and the functional annotation of ortholog groups

**DOI:** 10.1186/1471-2164-15-S6-S16

**Published:** 2014-10-17

**Authors:** Cécile Pereira, Alain Denise, Olivier Lespinet

**Affiliations:** 1 Univ Paris-Sud, Institut de Génétique et Microbiologie, UMR8621, 91405 Orsay, France; 2 Univ Paris-Sud, Laboratoire de Recherche en Informatique, UMR8623, 91405 Orsay, France; 3 CNRS, 91405 Orsay, France; 4 INRIA team AMIB, 91120 Palaiseau, France

**Keywords:** ortholog, homolog, meta-approach, sequences-profile comparison

## Abstract

**Background:**

In comparative genomics, orthologs are used to transfer annotation from genes already characterized to newly sequenced genomes. Many methods have been developed for finding orthologs in sets of genomes. However, the application of different methods on the same proteome set can lead to distinct orthology predictions.

**Methods:**

We developed a method based on a meta-approach that is able to combine the results of several methods for orthologous group prediction. The purpose of this method is to produce better quality results by using the overlapping results obtained from several individual orthologous gene prediction procedures. Our method proceeds in two steps. The first aims to construct seeds for groups of orthologous genes; these seeds correspond to the exact overlaps between the results of all or several methods. In the second step, these seed groups are expanded by using HMM profiles.

**Results:**

We evaluated our method on two standard reference benchmarks, OrthoBench and Orthology Benchmark Service. Our method presents a higher level of accurately predicted groups than the individual input methods of orthologous group prediction. Moreover, our method increases the number of annotated orthologous pairs without decreasing the annotation quality compared to twelve state-of-the-art methods.

**Conclusions:**

The meta-approach based method appears to be a reliable procedure for predicting orthologous groups. Since a large number of methods for predicting groups of orthologous genes exist, it is quite conceivable to apply this meta-approach to several combinations of different methods.

## Background

Performing an accurate gene/protein functional annotation is one of the crucial steps of any new genome project. It is partly achieved by performing the functional annotation of groups of orthologs.

Orthologs are genes in different species that arose from a common ancestral gene by speciation events [1]. Based on the 'orthology-function conjecture' [2,3], the orthologs retain the same function and thus can be used for the transfer of functional annotation from experimentally characterized genes to uncharacterized genes [4].

In this article, an ortholog group contains all the genes that evolved by gene duplication since the most ancestral speciation event of a given set of genomes [4]. Thus, ortholog groups include orthologs, co-orthologs and paralogs that evolved by lineage specific duplication after the relevant speciation event (in-paralogs) [5] (see Additional file 1).

The prediction of orthologous genes is a difficult task because of non-uniform evolutionary rates, extensive gene duplication, gene loss and horizontal gene transfer [6]. Over the last decades, a large number of methods and tools have been developed to perform orthologous gene prediction, and nowadays not less than 37 databases offer groups of orthologs [7]. However, the results predicted by these various methods are often uncertain. In particular, users should be aware that the application of different methods on the same proteomes can lead to distinct orthology predictions [6,8,9]. Accordingly to these results, it is particularly difficult to know which method or database will be the most appropriate. In addition, we might reasonably question the relevance of biological findings drawn from the orthology prediction obtained by any single method.

Sequence similarity is a good predictor of homology but does not define homolog sequences. Like the similarity used to predict homolog sequences, the genome context could be used to predict toporthologs (orthologous genes that retain their ancestral genomic position). This precision is motivated by the biological significance of genomic context [10] (genes that are near each other are more likely to interact [11] and are possibly coordinately expressed [12]). Because the gene order changes rapidly [13] and can not be use for distant species, we focus on the prediction of ortholog groups, without subdividing this groups into toporthologs and atoporthologs.

Prediction of gene orthology is based on two main approaches, namely tree-based methods and graph-based methods [14].

Tree-based methods are based on a tree-like evolutionary scenario and the evolution of the entire group of homologous genes is performed at the same time. The pairs of orthologs are inferred from the analysis of gene family trees and these methods [15-19] use the definition of orthology in order to distinct orthologs and paralogs. Gene orthology selection is generally done by tree reconciliation [20] with a reference species tree [17-19]. However, this last step becomes an issue when horizontal gene transfer plays a major role in the evolution of the organisms [21]. Moreover, tree-based methods are sensitive to artifacts, such as long and short-branch attraction at large or small evolutionary distances [22]. The results also depend of the quality of the species tree, which can contain errors especially at large evolutionary distances.

Graph based methods rely on the assumption that orthologous genes or proteins are more similar than any other gene or protein coming from the same organisms. Thus in graph based methods the orthologs are clustered together according to a similarity measure between the sequences. Several similarity scoring methods are used to cluster the sequences, for example BLAST derived scores [23] or similarity scores computed from Smith-Waterman alignments [24]. These methods [25-28] are generally much faster than tree-based methods and can deal with a larger number of species. However, they fail to detect differential gene losses [29,30] and can create mixed groups in the case of complex mixtures of differently-related genes.

As stated above, tree-based and graph-based methods have their advantages and drawbacks. In this work we propose to combine results obtained by several different methods by developing a meta-approach. The purpose is to produce better quality results by using the overlapping results obtained from several individual methods. The rationale behind our approach is that when identical results are found by several methods then they are more likely accurate. This is especially true as the prediction methods use different approaches like tree-based or graph-based methods. However, the overlap between multiple orthology prediction methods may lead to the loss of many true positives orthologs, especially when the number of initial methods is high. To overcome this problem the meta-approach is performed in two steps. An initial step finds seeds for groups of orthologous genes that correspond to the exact overlaps between all or at least several methods. In a second step we expand these seed groups by using HMM profiles.

Using acknowledged benchmark sets and procedures, we evaluated our metaapproach in relation to two aspects: the quality of our ortholog groups compared to known groups, and the relevance of functional sequence annotation based on our groups. The meta-approach allows to improve both.

## Methods

### The meta-approach

The entries of the meta-approach are ortholog groups obtained by several input methods. The general outline is as follows. First, we take into account only orthologs that are predicted by several methods, by selecting the intersections of their groups. From the sequences of the intersected groups, we build HMM profiles, possibly adding other sequences to the groups by comparing the sequences to the HMM profiles. Selection of the added sequences is based on the e-value and the percentage of alignment between the sequences and the HMM profiles. This whole process is performed several times, with the number of methods decreasing at each step, as detailed below.

At first, let us justify the meta-approach in a few words. It combines results from several methods, each of them having a given level of sensitivity and specificity. The first stage is stringent (specific), and tends to generate small orthologous groups, because each group is the intersection of the groups obtained by several methods. Recalling that our main objective at the end is annotation, what is important is not to have the largest possible groups, but to ensure that the genes that are in the same group will share the same function. From these small groups, which we call *intermediate groups*, HMM profiles are built. Proteins which are not in any intermediate group are called *unassigned proteins*. Each unassigned protein is compared to the profile HMM of each intermediate group and can be added to a group if the results of comparison satisfy conditions on the e-value and the minimum length of the alignment. Using the HMM profiles aims to improve the sensitivity of the results. Moreover, because the HMM profiles are made from several strongly selected protein sequences, we expect this step still to have a good specificity.

**Figure 1:**
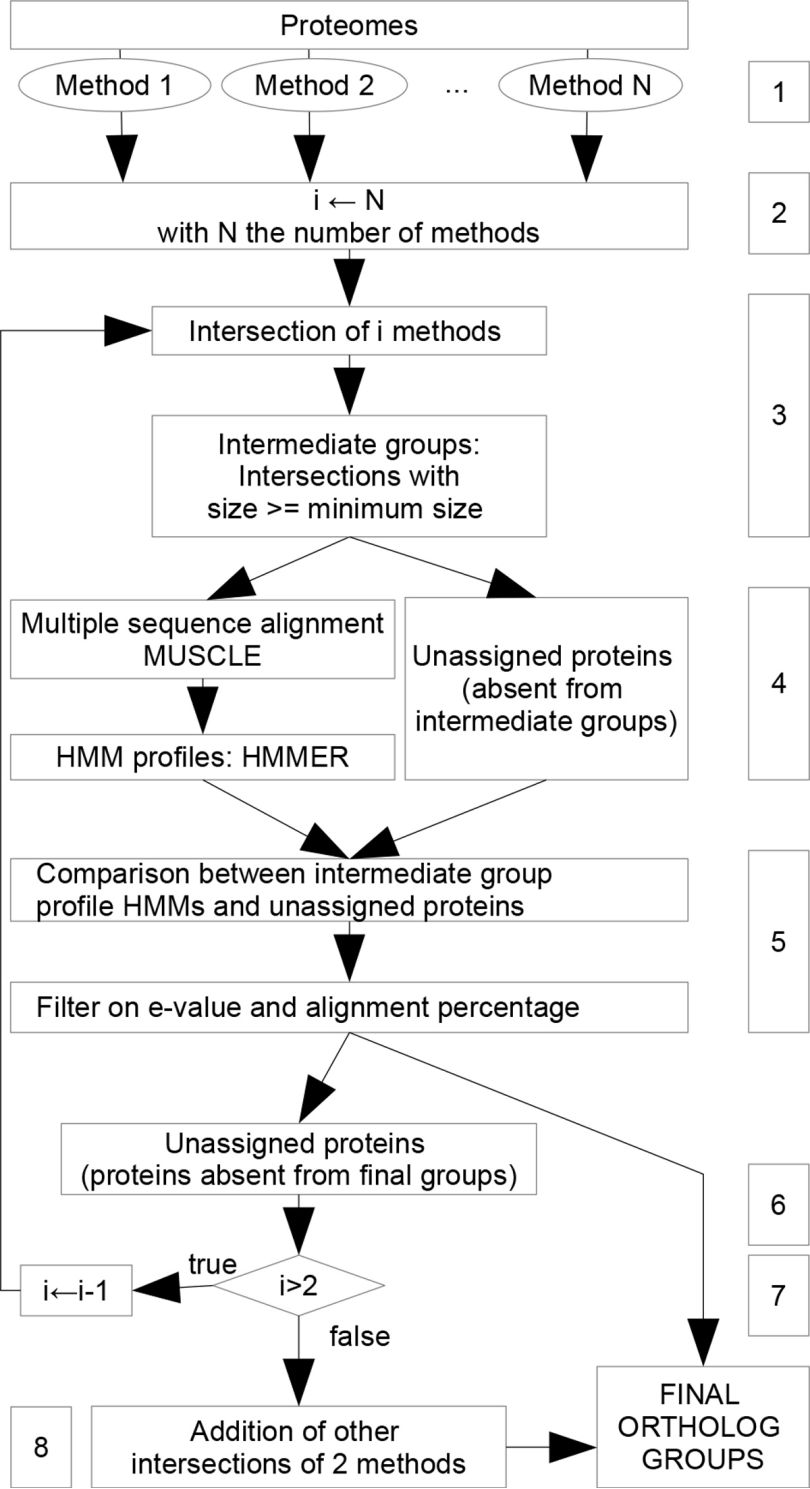
**Overview of the meta-approach**. The numbers in the boxes refer to the consecutive steps which are detailed in the text.

We present below the algorithm in more detail (see also Figure 1).

1 Collect ortholog groups from *N *input methods (*N *≥ 2)

2 set *i *= *N *.

3 Compute all sets of proteins that are intersections of groups of *i *methods: that is, two proteins are in the same set if and only if they are in the same group in *i *methods. Additionally, sets are selected according to their size: a set is selected only if its size is higher or equal to a given threshold (*minimum size *equals 4 as default). The selected sets form the intermediate groups. The proteins that do not belong to any intermediate group are called *unassigned proteins*. A protein cannot be in several intermediate groups. If this is the case, the largest intersection is kept (this occurs only when *i < N *). If there is some ambiguity for one protein (two distinct available groups of the same size) one of them is retained randomly.

4 For each intermediate group, a multiple alignment is generated with MUSCLE [31]. From each alignment, a profile HMM is computed using HMMER [32].

5 Each unassigned protein sequence is compared to each HMM profile. An unassigned protein is added to an intermediate group if: (i) the e-value of the comparison is lower than a given threshold (default 1E^−10^) and (ii) the length of the alignment is above a given ratio compared to the lengths of the sequence and of the profile (default 40%). An unassigned protein can be added to one intermediate group at most. If several HMMs satisfy the thresholds for the same unassigned protein, the lower e-value is retained, then the higher length ratio if necessary.

6 The groups obtained after the previous step are kept aside. This means that the proteins contained in these groups are not used for the next steps. They will be final ortholog groups.

7 If *i >*2 and if there still some unassigned proteins, then *i *← *i *− 1 and GOTO step 3.

8 Otherwise (*i *= 2), the loop stops. There can remain intersection groups that have not been selected as intermediate groups because their cardinality is smaller than the *minimum size*. These groups are added to the final ortholog groups (note that these are necessarily results of intersections of two methods only.)

The values of the three parameters (e-value, minimum length of the alignment and minimum intersection size) were determined by comparing results obtained with different parameter combinations from the same data set (see Additional file 2).

#### Sotware availability

The MARIO software which implements the meta-approach is freely available at http://bim.igmors.u-psud.fr/mario/.

### The selected input methods

The meta-approach was performed by using the results of four methods (BRH [33], Inparanoid [26], OrthoMCL [25] and Phylogeny [34]). The three graph based methods that we selected (BRH, Inparanoid, OrthoMCL) present distinct approaches for predicting ortholog pairs and then for producing groups. They are among the most representative of graph-based methods. A method developed previously in our laboratory called 'Phylogeny' was used as a representative of tree-based methods. All these methods have been implemented in stand-alone programs.

The initial Best Reciprocal Hit (BRH) method [33] was modified by taking into account the sequence alignment length as well as the alignment score ratio between query and subject sequences. The score ratio is the ratio of the raw BLAST score of the alignment and the raw score of each sequence against itself. All pairs of best reciprocal hits *i.e*. were both filters are above the threshold values are considered as orthologs. Pairs of orthologs are clustered by identifying fully connected orthologous groups: each protein of any given ortholog group has an orthology relationship with every other protein in the group (in our case, searching for such cliques is computationally tractable because, in the BRH method, each group presents up to one protein per species). Inparanoid [26] was used with default parameters. This method predicts pairs of orthologs and inparalogs. The pairs are clustered into groups in such a way that each protein of any group is linked to at least 20% of the other proteins of the group. OrthoMCL [25] uses the percentage match length to obtain pairs of orthologous proteins. The method clusters the pairs into groups by using the MCL program [35]. OrthoMCL was used with default parameters. Phylogeny [34] is based on the phylogenetic analysis of homologous genes. No species tree is required. Homologs detected by BLAST are grouped transitively. Homologous sequences are aligned using the MUSCLE program. These multiple alignments are processed with a maximum likelihood approach to reconstruct the phylogeny of the corresponding family, using the PhyML software. Group trees are rooted by using the program Retree from the Phylip package [36]. The analysis of the rooted tree allows to identify duplication and speciation events and to distinguish orthologs and paralogs.

### Evaluation

In order to evaluate our meta-approach, we checked its consistency according to the ability to predict ortholog groups, and the quality of protein functional annotation within an ortholog group. We used two benchmarks: OrthoBench and the Orthology Benchmark Service. The values of the parameters used on both benchmark tests for the meta-approach were the same, as stated above: minimum e-value 1E*^−^*10, minimum alignment length of 40%, minimum intersection size equal to four.

#### Evaluation on 70 reference ortholog groups

Taking orthoBENCH [37] as a reference benchmark, we compared the results of the four initial methods, and those obtained by the meta-approach, to the reference ortholog groups (RefOGs). The orthoBENCH dataset involves 1519 proteins from 12 metazoan species divided into 70 manually curated ortholog groups. For our analysis, we downloaded the proteome version of Ensembl 72 [38]. As orthoBENCH is based on Ensembl 62, the proteins removed or added between the versions 60 and 72 of Ensembl were not taken into account. Moreover, if a gene has splice variants, When comparing the groups produced by the meta-approach or the individual methods with those of orthoBench, two types of errors were defined: group fissions (proteins of a RefOG are in two or more ortholog groups), and group fusions (more than 3 proteins have been added to a RefOG) [37].

#### Functional annotation conservation test

The Orthology Benchmark Service is a recent web server (http://orthology.benchmarkservice.org) allowing us to compare methods of orthologous gene prediction. This is based on a common set of 66 species (2011 quest for orthologs reference dataset) [39,40]. The benchmark service proposes two types of procedures for evaluating orthologous groups: phylogeny-based definition tests and functional annotation conservation test. In the Phylogeny based tests, orthologous groups are defined in such a way that every pair of genes in the group is either orthologous or inparalogous with respect to the last speciation event in their clade. However, we refer to a different and more recent definition of ortholog groups [4]. Thus this test is not relevant for our purpose (see Additional file 1 for further details).

The web server proposes also evaluation procedures for measuring the homogeneity of the functional annotation of the pairs of orthologs [7]. For each pair, if both proteins are annotated, the similarity of the annotation is computed with the Schlicker similarity [41]. This measure allows partial matches, resulting in a robust similarity score for the comparison of gene products with incomplete annotation or for the comparison of multi-functional proteins. This score ranges between 0 and 1, from low to high functional similarity. We computed this measure for Enzyme Commission (EC) numbers [42] and for Gene Ontology (GO) terms [43]. For GO terms, only annotations with experimental support (EXP, IDA, IPI, IMP, IGI and IEP) were considered.

## Results and discussion

At first we briefly present the results of the four initial methods and of the metaapproach on the orthoBENCH dataset. Then we compare the meta-approach to twelve other state-of-the-art methods from the functional similarity point of view.

### Comparing the results of the four initial methods with those of the meta-approach

The results obtained for each of the four initial methods were compared with those obtained by the meta-approach (Table 1 and see Additional file 3) on the orthoBENCH dataset. First we observe that all the methods produce different numbers of ortholog groups (ranging from 14 771 for the meta-approach to 25 384 for BRH), and, in addition predict orthology relationships for different numbers of proteins. In order to measure the similarity between the groups obtained by each method, we computed the Jaccard coefficient by dividing the number of ortholog pairs in common between two methods by the total number of pairs of orthologs (*|A *∩ *B|/|A *∪ *B|*). The Jaccard coefficient value is expected to be between 0 (no or-tholog pairs in common) and 1 (all couples are identical). In our case, all the values range from 0.164 to 0.541. This means that all the methods individually produce rather different results. The Jaccard coefficient values between the meta-approach and any of the input methods are even lower (lower than 0.156). In other words, none of the selected methods alone can explain the result of the meta-approach.

**Table 1:** Comparison on OrthoBENCH [37], Jaccard similarity coefficient.

		BRH	**Inp**.	**Ort**.	**Phy**.	Meta
Jaccard	BRH		0.541	0.172	0.389	0.060
similarity	Inp.			0.248	0.340	0.093
coefficient	Ort.				0.164	0.156
	Phy.					0.079
#Proteins		140561	163850	155982	124206	187902

Abbreviations : 'Meta' refers to Meta-approach, 'Phy.' to phylogeny, 'Inp.' to inparanoid and 'Ort.' to orthoMCL.

#### Quality of ortholog groups

**Figure 2:**
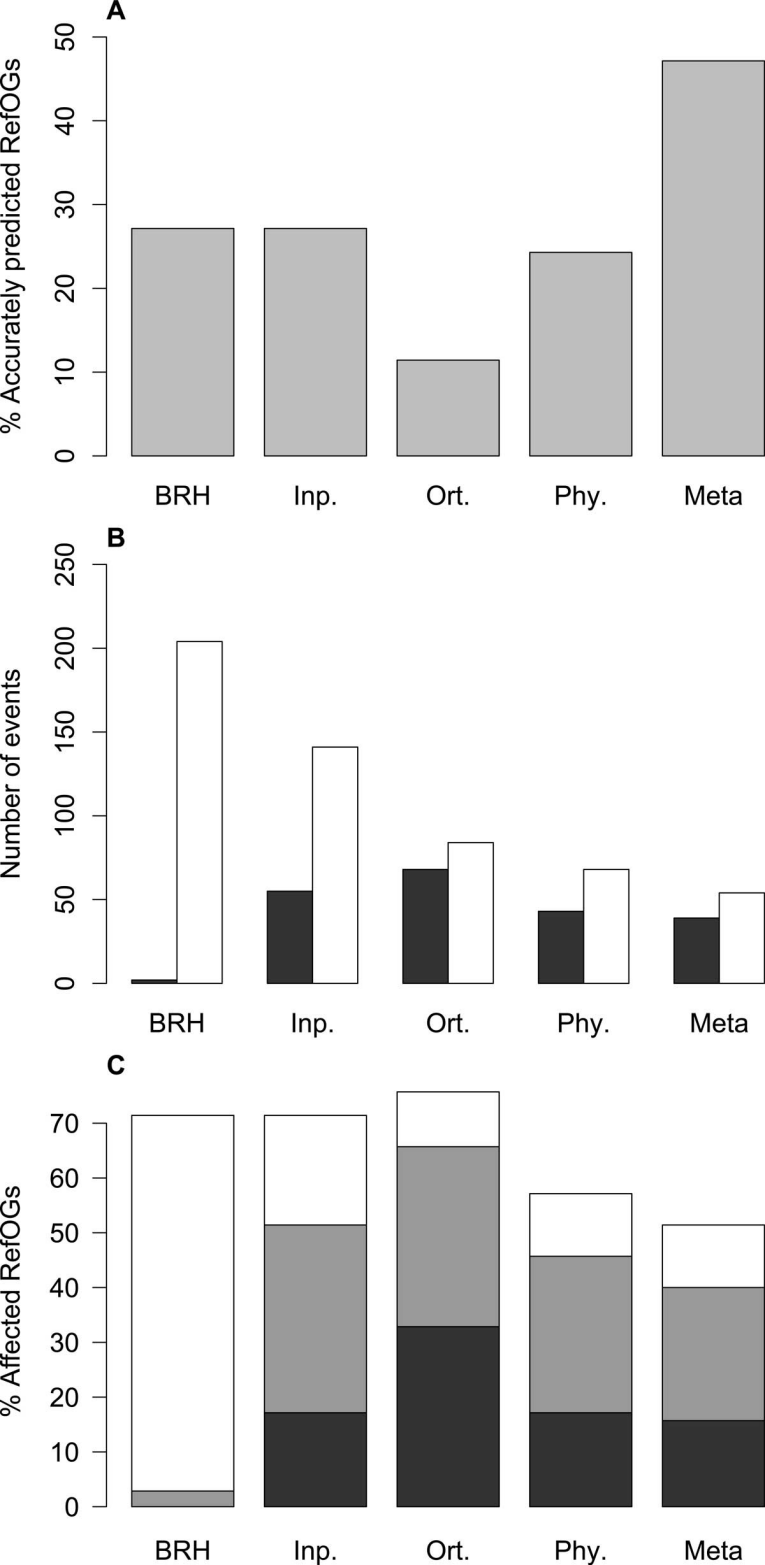
**Comparison of the predicted ortholog groups quality (benchmark OrthoBENCH)**. (A) Percentage of accurately predicted RefOGs (groups predicted without fusion or fission events), (B) Number of fusions (in dark gray) or fissions (in white), (C) Percentage of RefOGs affected by a fusion event (in dark gray), by a fission event (in white) or by the booth (in light gray). A fusion of groups corresponds to the addition of more than 3 erroneously assigned genes to a RefOG. Fissions correspond to a RefOG split in several groups: *n *group gives *n *− 1 fissions. Abbreviations: 'Meta' refers to Meta-approach, 'BRH' to BRH [33], 'Phy.' to Phylogeny [34], 'Inp.' to Inparanoid [26] and 'Ort.' to orthoMCL [25].

Among the four input methods, BRH and Inparanoid give the highest level of accurately predicted groups (groups without fusion or fission events) (Figure 2A). BRH presents the highest number of fissions and the smallest number of fusions (Figure 2B). Inparanoid allows the detection of in-paralogs between each pair of proteomes and thus the number of fusions is higher than with BRH and Phylogeny. The Phylogeny approach presents the smallest number of groups impacted by fusions or fissions. The OrthoMCL method presents groups largely impacted by fusion events compared with the other three methods. The larger number of fusions is associated to a lower number of fissions. This result on orthoMCL is consistent with the results obtained by Dalquen *et al *[6] on a dataset of mammalian genomes. As for the meta-approach, it presents the lowest percentage of groups affected by fission or fusion events (Figure 2C). It also allows an increase of 73.7% in the number of accurately predicted groups compared to the highest result obtained with the four initial methods (Figure 2A). At the same time, the meta-approach presents the lowest number of fissions and a number of fusions lower than three of the initial methods alone (Figure 2B). This demonstrate that the meta-approach improves the results obtained with any of the initial methods, either graph-based or tree-based.

### Functional similarity performance comparison

We compared twelve methods including all those available in the orthology benchmark service and the four selected input methods for the analysis of the reference proteomes [40]. Additionally, in order to evaluate the impact of using HMM on a single method, we applied the profile HMM procedure (steps 4, 5 and 8) to the BRH groups. For the meta-approach, we used the same values of parameters as those used for the orthoBENCH analyses.

#### Enzyme classification conservation test

**Figure 3:**
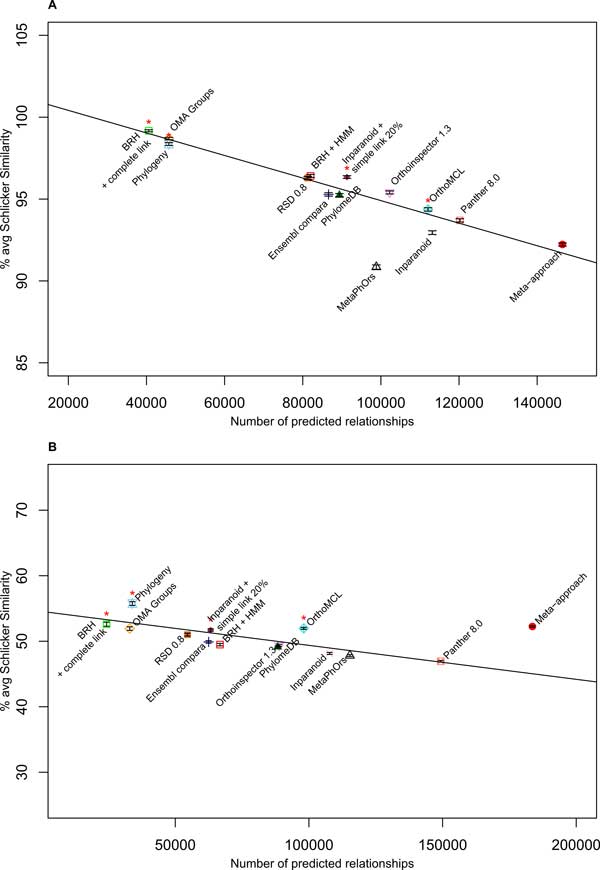
**Function-Based Tests**. (A) Enzyme classification conservation test. The linear regression curve has an intercept value of 101.8 and a regression coefficient of −6.887*E*−05. (B) Gene ontology conservation test. The linear regression curve obtained on the GO term annotation has an intercept value of 54,55 and a regression coefficient of −5.184*E*−05. The black lines are the linear regression obtained on all methods except the meta-approach, the metaPhors and the BRH plus HMM profiles approach. Error bars for each method are in black. Fourteen methods were compared: *·*: Meta-approach. Δ: metaPhOrs [18]. □: BRH [33] with complete link. □: BRH with complete link plus HMM steps. +: Ensembl compara v2 [38]. ×: Inparanoid pairs [26]. œ: OMA Groups [27]. ∇: orthoinspector 1.30 [44]. ⊠: PANTHER 8.0 [45]. ▲: phylomeDB [19]. ■: Roundup (RSD 0.8) [28]. ◆: Inparanoid with 20% simple link. œ: OrthoMCL. ⋆: Phylogeny. ✱: The four methods used for the meta-approach.

The Pearson correlation test was performed with and without the results of the meta-approach in order to determine the relationship between the number of annotated orthologs and the average Schlicker similarity obtained with the EC number annotations. The results of the metaPhors [18] method stored on the orthology benchmark service website was not available for all the species, therefore this approach was not used for the calculation of correlation. The Pearson correlation is significant whether we use results of the meta-approach or not. The Pearson correlation equals −0.971 (p-value 7.436*E*−8) using the meta-approach and, −0.964 (p-value 8.573*E*−7) without the meta-approach (negative correlation hypothesis). This means that increasing the number of ortholog relations is correlated with a decrease in the average Schlicker similarity (Figure 3A). All methods present a percentage of Schlicker similarity higher than 90%, revealing that all methods succeed in predicting pairs of enzymes with a similar function. Finally, the meta-approach also finds the largest number of ortholog relationships.

#### Gene ontology conservation test

The Pearson correlation test was performed without taking into account the metaapproach in order to determine the relationship between the number of annotated orthologs and the average Schlicker similarity obtained on GO terms. The results of the metaPhors [18] method were not used for the same reason as indicated previously. The Pearson coefficient was -0.804 (p-value 1.419*E*−3 with the negative correlation hypothesis). Thus, as for the EC number similarity, the larger number of ortholog relations is correlated to the decreasing of the average Schlicker similarity. The meta-approach detects an increased number of ortholog relations compared to orther methods (Figure 3B). The Pearson correlation test was performed on the results of all methods (the meta-approach plus the twelve others) in order to determine if the meta-appraoch presents results that are compatible with the same linear regression curve as obtained with the other methods. The Pearson coefficient is not significant (−0.471 and p-value 0.06121 with the negative correlation hypothesis), showing that the result obtained with the meta-approach is not compatible with the linear regression. Furthermore, the point representing the meta-approach is above the linear regression curve (Figure 3B), showing that the meta-approach outperforms the other methods on this dataset. Thus, the meta-approach increases both the average Schlicker similarity and the number of ortholog relationships. In addition, the application of the HMM steps on the BRH groups increases the number of annotated ortholog pairs. However and contrary to the meta-approach, the Schlicker similarity decreases when the number of ortholog relation increase, as predicted by the linear regression. Therefore, the combination of the results of several methods is necessary to improve the quality of the final prediction.

## Conclusions

The meta-approach appears to be a reliable method of prediction of ortholog groups. Based on the combination of existing methods, the meta-approach finds a consensus of higher quality. Both ortholog group quality and consistence of group annotation have been positively tested. We showed with the orthoBench dataset [37] that, compared to the initial methods, the meta-approach reduce the number of incorrect groups as well as the number of fission and fusion events. Furthermore, the metaapproach presents the largest GO term similarity compared to twelve of the thirteen state-of-the-art methods on the protein reference dataset [40]. Phylogeny's Schlicker similarity is larger than the meta-approach, but Phylogeny predicts many less pairs of annotated orthologs. All other methods present both a smaller Schlicker similarity and an smaller number of pairs of annotated orthologs.

The meta-approach combines the results of several methods in order to obtain specific intersections and adds to these intersections similar sequences (by using profile HMMs). The user has to be well aware that results depend of the selected input methods and on the selected parameters for the HMM profiles.

The meta-approach presented in this article takes the benefits from the particular four methods used here, but as a large number of methods for predicting groups of orthologous genes exist, it would be interesting to apply this meta-approach to different methods or to more methods.

## Competing interests

The authors declare that they have no competing interests.

## Authors' contributions

CP developed and tested the meta-approach. OL and AD supervised the work. All authors (CP, AD, and OL) drafted, read and approved the final manuscript.

## Supplementary Material

Additional file 1 **Comparison of group trees obtained with two definitions of ortholog groups**. The phylogeny-based definition tests select ortholog groups in which at least one protein of each of the *n *species is present. If several proteins are available, one of them is selected randomly, which can lead to differences between the species tree topology and the gene tree topology depending on the ortholog group definition. (A) Example of a specie tree with four species. Each speciation event is presented by a 'S' and a number associated. (B) Possibles associated gene trees and ortholog groups. Green : ortholog group at the S1 level, pink: ortholog group with in-paralogs allowed only if the duplication occurred after the last speciation event (phylogeny tree test definition). Stars: duplication events. (C) Gene trees possibly evaluates with the phylogenetic tree test. This gene trees results from the random selection of one sequence of each species from the ortholog group at the S1 level (green) presented in sub-figure B. In grey, gene tree inducing high Robinson-Foulds distance while the ortholog group is coherent at the S1 level. The larger the number of species used and the more this type of error will occur. Click here for file

Additional file 2 **The impact of each parameter of the meta-approach evaluated on orthoBench**. Each column (1,2,3) corresponds to the evaluation of a parameter. Like in the Figure 2, the graphs (A) corresponds to the percentage of accurately predicted RefOGs, graphs (B) corresponds to the number of fusions (in dark gray) or fissions (in white) and graphs (C) corresponds to the percentage of RefOGs affected by a fusion event (in dark gray) or by a fission event (in white) or by both types of events (in light gray). The impact of the e-value threshold is observed in the column 1. The two other parameters are fixed (minimum alignment length of 40% and minimum intersection size of six). The variation of the e-value does not involve a large variation in the quality of the predicted groups. The selected value is 1*E*−10 (highest accurately predicted RefOGs and smallest percentage of groups affected by fission or fusion events). The impact of the minimum alignment length parameter (used in step 5 of the meta-approach) is observed in the column 2. The e-value is fixed to 1*E*−5 and the minimum intersection size equal three. According to this chart, the increase of the required alignment induces the decrease of the number of fusion and the increase of the number of fission. The highest accurately predicted RefOGs is obtained with the values 40% and 80%. The impact of the minimum size parameter (used in step 3 of the meta-approach) is observed on the column 3. The two other parameters are 1*E*−5 for the e-value and 40% for the minimum alignment length. Results obtained with intersections of size four or more presents the highest number of accurately predicted groups. However, this evaluation was performed on only 12 species. Thus, the number of ortholog groups containing more than 4 sequences could have induce an under-evaluation of the value of this parameter. Click here for file

Additional file 3 **Identical groups on OrthoBENCH**. Number of identical groups finds on OrthoBENCH for every pair of methods. Click here for file

## Declarations

The publication charges for this article were funded by CNRS-INSERM-INRIA grant PEPS Bio-Math-Info (BMI) 2012-2013.

This article has been published as part of *BMC Genomics *Volume 15 Supplement 6, 2014: Proceedings of the Twelfth Annual Research in Computational Molecular Biology (RECOMB) Satellite Workshop on Comparative Genomics. The full contents of the supplement are available online at http://www.biomedcentral.com/bmcgenomics/supplements/15/S6.
