# Supplementary figures and images for: A meta-approach for improving the prediction and the functional annotation of ortholog groups
Source: BMC Genomics. 2014 Oct 17;15(Suppl 6):S16. doi: 10.1186/1471-2164-15-S6-S16 (PMC4240552; doi:10.1186/1471-2164-15-S6-S16)

**A**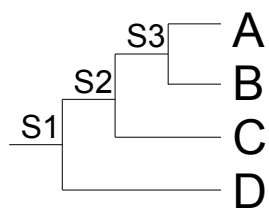**B**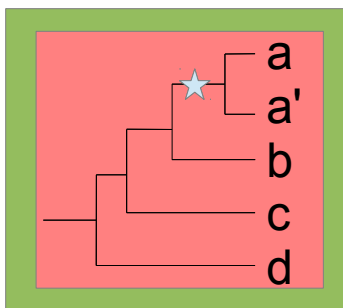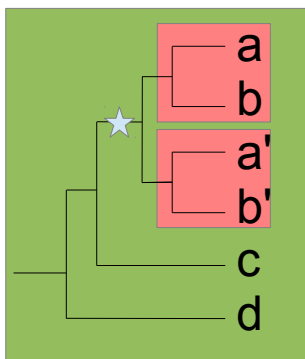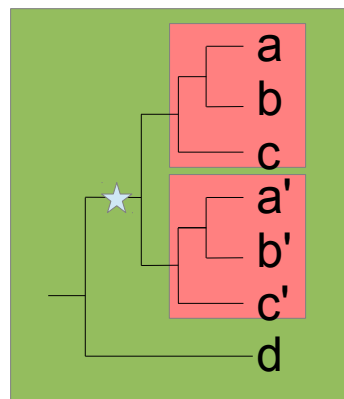**C**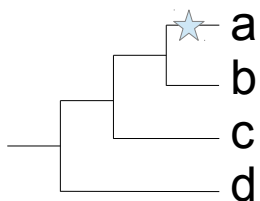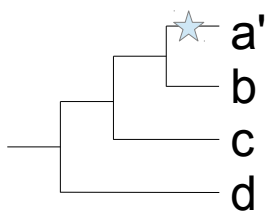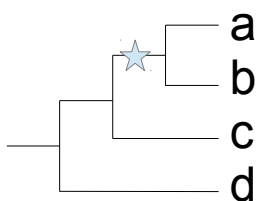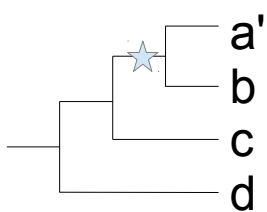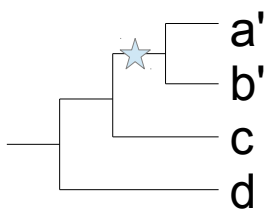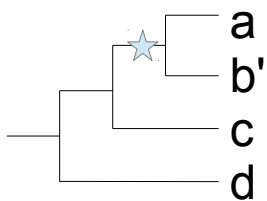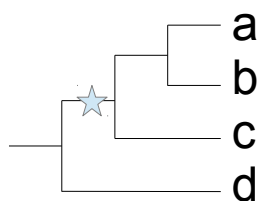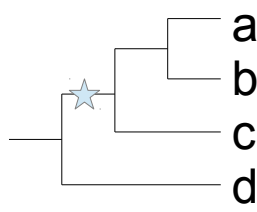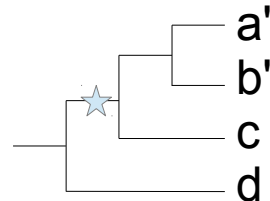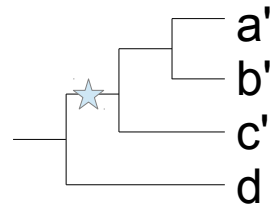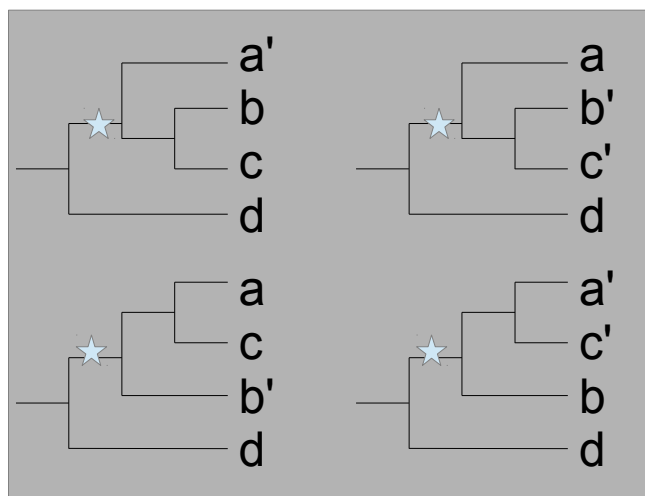

Supplement: Additional file 1 — Comparison of group trees obtained with two definitions of ortholog groups. The phylogeny-based definition tests select ortholog groups in which at least one protein of each of the n species is present. If several proteins are available, one of them is selected randomly, which can lead to differences between the species tree topology and the gene tree topology depending on the ortholog group definition. (A) Example of a specie tree with four species. Each speciation event is presented by a 'S' and a number associated. (B) Possibles associated gene trees and ortholog groups. Green : ortholog group at the S1 level, pink: ortholog group with in-paralogs allowed only if the duplication occurred after the last speciation event (phylogeny tree test definition). Stars: duplication events. (C) Gene trees possibly evaluates with the phylogenetic tree test. This gene trees results from the random selection of one sequence of each species from the ortholog group at the S1 level (green) presented in sub-figure B. In grey, gene tree inducing high Robinson-Foulds distance while the ortholog group is coherent at the S1 level. The larger the number of species used and the more this type of error will occur. [file 1471-2164-15-S6-S16-S1.pdf]
