# Supplementary material for: A meta-approach for improving the prediction and the functional annotation of ortholog groups
Source: BMC Genomics. 2014 Oct 17;15(Suppl 6):S16. doi: 10.1186/1471-2164-15-S6-S16 (PMC4240552; doi:10.1186/1471-2164-15-S6-S16)
Supplement: Additional file 3 — Identical groups on OrthoBENCH. Number of identical groups finds on OrthoBENCH for every pair of methods. [file 1471-2164-15-S6-S16-S3.pdf]

		BRH	Inparanoid	OrthoMCL	Phylogeny	Meta-approach
# Identical groups	BRH	25384				
	Inparanoid	7543	21342			
	OrthoMCL	5272	7268	17524		
	Phylogeny	4082	3260	2696	17944	
	Meta-approach	3322	4705	4463	2654	14771
# Proteins		140561	163850	155982	124206	187902
